# The Beginner’s Guide to *O*-GlcNAc: From Nutrient Sensitive Pathway Regulation to Its Impact on the Immune System

**DOI:** 10.3389/fimmu.2022.828648

**Published:** 2022-01-31

**Authors:** Michael P. Mannino, Gerald W. Hart

**Affiliations:** Complex Carbohydrate Research Center, University of Georgia Athens, Athens, GA, United States

**Keywords:** GlcNAc, immune system, post translational modification, protein-protein interactions, glycobiology, nutrient sensing, cell signaling, lymphocyte activation

## Abstract

The addition of N-acetyl glucosamine (GlcNAc) on the hydroxy group of serine/threonine residues is known as *O*-GlcNAcylation (OGN). The dynamic cycling of this monosaccharide on and off substrates occurs *via O*-linked β-N-acetylglucosamine transferase (OGT) and *O*-linked β-N-acetylglucosaminase (OGA) respectively. These enzymes are found ubiquitously in eukaryotes and genetic knock outs of the *ogt* gene has been found to be lethal in embryonic mice. The substrate scope of these enzymes is vast, over 15,000 proteins across 43 species have been identified with *O*-GlcNAc. OGN has been known to play a key role in several cellular processes such as: transcription, translation, cell signaling, nutrient sensing, immune cell development and various steps of the cell cycle. However, its dysregulation is present in various diseases: cancer, neurodegenerative diseases, diabetes. *O*-GlcNAc is heavily involved in cross talk with other post-translational modifications (PTM), such as phosphorylation, acetylation, and ubiquitination, by regulating each other’s cycling enzymes or directly competing addition on the same substrate. This crosstalk between PTMs can affect gene expression, protein localization, and protein stability; therefore, regulating a multitude of cell signaling pathways. In this review the roles of OGN will be discussed. The effect *O*-GlcNAc exerts over protein-protein interactions, the various forms of crosstalk with other PTMs, and its role as a nutrient sensor will be highlighted. A summary of how these *O*-GlcNAc driven processes effect the immune system will also be included.

## Introduction

*O*-N-acetylglucosamine (*O*-GlcNAc) is a monosaccharide, post translational modification (PTM) covalently bound to serine and/or threonine residues. Unlike traditional forms of glycosylation, the addition of a *O*-GlcNAc moiety, *O*-GlcNAcylation, occurs within nucleocytoplasmic and mitochondrial compartments of the cell and remains as a monosaccharide opposed to further elaboration to a polysaccharide (1, 2). Similar to other PTMs like phosphorylation, *O*-GlcNAc is cycled on and off substrates, regulating their biological functions. However, unlike phosphorylation, the addition and removal of this modification is performed with a single set of enzymes, *O-β*-N-acetylglucosamine transferase (OGT) and *O-β*-N-acetylglucosamidase (OGA) respectively (3, 4).

These enzymes are found primarily in the nucleus, cytoplasm, and mitochondria in all metazoans, including plants viruses, and some bacteria (5, 6). Deletion of OGT or OGA is embryonically and perinatally lethal in mice, demonstrating its importance to survival and development (7, 8). The number of identified *O*-GlcNAcylated substrates is continuously growing at a rapid pace thanks to technological advances. Perhaps due to its ubiquitous nature and biological importance, various *O*-GlcNAc databases have been developed to mainstream pertinent information regarding *O*-GlcNAcylated substrates, such as corresponding literature references and, in some cases, the amino acid site of the modification (9, 10).

*O*-GlcNAcylation plays a role in a broad range of biological processes, such as transcription, translation, enzyme activity, cell division, protein localization and degradation. How these and other cellular operations are regulated is, in part, dependent on which substrates are *O*-GlcNAcylated and to what extent. As will be discussed later in more detail, OGT’s activity and substrate specificity vary with the concentration of UDP-GlcNAc within cells (11), the donor for *O*-GlcNAc, which is proportional to the flux of several metabolites (12–15). Thus, *O*-GlcNAc directly links the regulation of important biological processes with the cellular nutrient status to serve as a major nutrient sensor. This is further highlighted in metabolic diseases, such as diabetes and cancer, whose aberrant *O*-GlcNAc levels are correlated with pathologic phenotypes (16–20).

## *O*-GlcNAc Cycling Enzymes

### *O*-GlcNAc Transferase (OGT)

The *ogt* gene is highly conserved in numerous organisms from C. elegans to humans and is encoded on the X chromosome near the centromere, exhibiting greater than 60-80% amino acid identity between species (8). OGT was initially purified from rat liver and reticulocyte lysates (3, 21), sequenced and cloned (22, 23). The tertiary structure of the enzyme was determined in 2011 by overlapping two semi-complete crystal structures, the breakthrough of which contained a UDP and CKII peptide molecules bound to the active site (24). Belonging to the GT-B superfamily of glycosyltransferases, OGT is made up of four domains: 1) a N-terminal domain containing continuous helix-turn-helix tetratricopeptide repeats (TPRs), 2) C-terminal region bearing the GT41 catalytic domain containing two Rossmann-folded lobes, 3) an intervening region that bridges the two lobes and 4) a nuclear localization sequence between the TPR and catalytic domain (25). OGT has three different isoforms, all varying in the length of their respective TPR domains. ncOGT (nuclear cytoplasmic) and sOGT (short) have 13.5 and 2.5 TPRs, both of which are found in the cytoplasm and nucleus. mOGT (mitochondria) consists of 9 TPR domains and is localized in the mitochondrial inner membrane (26). ncOGT is the predominant isoform (27).

Much work has been done to elucidate OGT’s binding modes and substrate specificity, most of which is done through crystallization and related mutagenesis studies. One of the earliest was a crystal structure of OGT with the non-hydrolyzable GlcNAc derivative and OGT inhibiter UDP-5SGlcNAc, which identified an ordered bi-bi mechanism of glycosylation where UDP-GlcNAc initially binds to the active site and then is covered by the acceptor (28). Although there is one example of substrate binding to the catalytic domain alone (29), the majority of substrate binding is suggested to occur in the TPR domain of OGT. The removal of the TPR domain is known to abrogate OGT activity toward protein substrates but not to small peptide substrates (30, 31). Interestingly, unlike other small peptides, OGT does not modify the C-terminal domain (CTD) of RNA polymerase II, which consists of a degenerate seven amino acid repeats, if it has less than five repeats (35 amino acids). However, ten CTD repeats is an excellent substrate *in vitro* (32). Key structural features of the TPR domain have been identified thanks to crystal structures of various substrate-bound OGT complexes. The TPR domain forms a superhelix made up of two layers, the inner layer contains a highly conserved asparagine ladder which binds the amide backbone of the acceptor substrates (33). This ladder extends the length of the TPR region, the mutations of which were shown to impair protein *O*-GlcNAcylation (24, 34). Recent contributions in the literature have revealed the presence of two aspartate residues along the inner layer of the TPR domain proximal to the active site, the alanine mutation of which diminishes substrate binding (35). In this study and several earlier ones, an attempt to determine a consensus substrate sequence was attempted (3, 36, 37). From these investigations it is suggested that about half of the known OGT substrates contain acidic arginine or lysine residues within 7-11 amino acids of the functionalized threonine or serine.

Given these statistical findings, it has largely been agreed upon that there is no consensus sequence for OGT substrates. This may in part be due to OGT’s employment of adapter proteins. These are proteins that form complexes with OGT and subsequently direct substrate specificity by altering binding modes or localization. Various adapter proteins, such as mSin3a, PCG-1a and HDAC1 have been identified (38, 39), the most studied are OGT’s interactions and targeting by ten-eleven translocation (TET) family enzymes. OGT-TET complexes target OGT to chromatin or histones that are involved in chromatin remodeling (40). Although adapter protein identification and significance can be difficult to determine, recent advancements in labeling technology utilizing a biotin transferase-TPR fusion protein may provide additional examples of proteins acting in this manner (41). Expanding the list of known adapter proteins and their specific functions will help explain the promiscuity and specificity of OGT.

### *O*-GlcNAcase (OGA)

The *oga* gene (*mgea5)*, which was initially identified as a putative hyaluronidase and cloned from a meningioma, is present on the somatic chromosome 10. OGA protein was first purified from rat spleen (4) and rat brain (42). The rat brain OGA was sequenced and cloned and it was found to be identical to *mgea5*. OGA protein exists in two isoforms, the predominant 916 amino acid OGA-L and the less common short OGA-S truncated in the C-terminus. OGA consists of three domains: 1) a N-terminal catalytic domain, similar to glycoside hydrolase family 84 (GH84) enzymes with a [(β/α)_8_] triose-phosphate isomerase (TIM) barrel structure, 2) a stalk region or α-helical bundle, and 3) a C-terminal pseudo-histone acetyltransferase (HAT) domain which does not have any acetyl transferase activity (42).

Several crystallization studies in 2017 have elucidated various aspects of OGA’s structure (43, 44). These studies indicate that human OGA forms homo dimmers with a single stalk α-helix of the opposing monomer. Li and coworkers showed that removing one of the stalk helices exhibited 100-fold lower catalytic activity. OGA is cleaved in half by caspase 3 during apoptosis, but the enzyme remains active and the catalytic domain and HAT domains remain non-covalently associated (45). Two conserved aspartate residues were found in OGA’s active site and binding pocket flanking the *O*-GlcNAc glycosidic bond, potentially catalyzing its hydrolysis (43, 46). The OGA dimer forms a V-shaped cleft at the interface of the catalytic domain and stalk domain of the two monomers, providing a potential substrate binding pocket. For a more detailed discussion on OGT and OGA substrate specificity see (47).

## *O*-GlcNAc Regulates Protein Function

In the first two and a half decades since the discovery of intracellular *O*-GlcNAc, around 500 *O*-GlcNAcylated proteins had been reported in the literature (48). Since then, advancements in analytical methods, specifically mass spectrometry and labeling techniques, have simplified and fast-tracked the elucidation of the *O*-GlcNAcome (49–52). Recently, multiple databases have been constructed mainstreaming the search for *O*-GlcNAcylated targets and even providing site-specific mapping when available (9, 10). As of November 2021, according to the https://www.oglcnac.mcw.edu/statistics/, there are over 15,000 GlcNAcylated proteins from 43 different species reported in the literature curated from around 2,300 articles. The role of these *O*-GlcNAcylated substrates extends to nearly every intracellular biological process imaginable: cell metabolism (53, 54), cell death (55), the circadian clock (56–58), cell cycle progression (13), various signaling pathways (59, 60), transcription (61), translation (62, 63), protein degradation (64), and cell development (65). Like other PTMs, such as phosphorylation, *O*-GlcNAc’s cycling conveys a bevy of biological outcomes by modifying the substrate’s function. A brief overview of these *O*-GlcNAc modified activities will be discussed below, however for a more complete discussion please see the references (53, 66).

**Figure 1:**
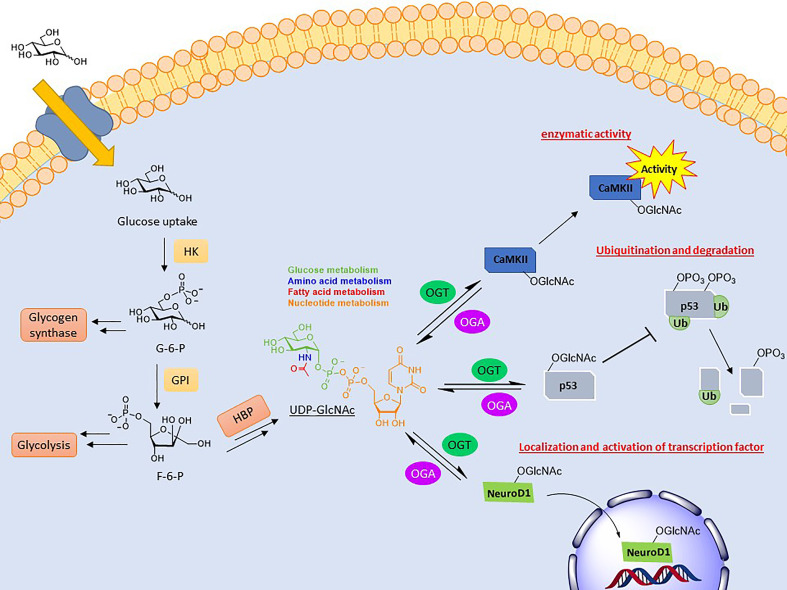
Depiction of the biosynthesis of UDP-GlcNAc and the effects conveyed on its substrates. UDP-GlcNAc is formed through the hexosamine biosynthetic pathway (HBP), combining several metabolites. *O*-GlcNAc cycling can modulate activity (CaMKII), stability (p53), and localization (NeuroD1) of its substrates.

A common result of *O*-GlcNAcylation is the relocalization of its substrate. This effect is most often observed with transcription factors, resulting in their activation or inhibition. NeuroD1 is a transcription factor in pancreatic β-cells which induces gene expression responsible for insulin production (67). Upon *O*-GlcNAcylation, this cytosolic protein is localized to the nucleus to initiate transcription (**Figure 1**). This was determined in the context of elevated glucose concentrations driving NeuroD1 nuclear translocation, which was ameliorated upon *ogt* silencing *via* siRNA (68). *O*-GlcNAcylation of β-catenin has the opposite effect. Modification at Ser23 was shown to induce plasma membrane localization, where it activates cell adhesion functionality, consequently blocking its nuclear localization and gene transcription (69).

Another key function of *O*-GlcNAc cycling is the inhibition or activation of enzymes. Calcium/calmodulin-dependent protein kinase II (CaMKII) is a regulatory kinase involved in Ca^2+^ release events important for heart and brain function. Hyperglycemia is correlated with the chronic activation of this protein, which has been shown to induce arrhythmias and other cardiomyopathic phenotypes (70). Elevated *O*-GlcNAcylation of this protein, specifically at S279, was shown to induce autonomous activation of CaMKII under hyperglycemic conditions even in the absence of Ca^2+^/calmodulin (**Figure 1**). FRET analysis demonstrated that this activation is perpetuated by inhibiting its reversion to an apo-conformation. Mutation of this site attenuated these effects (71).

The previous example also demonstrates how *O*-GlcNAcylation can alter substrate conformation and consequently its function. A more indirect example is the modification’s influence on heat shock proteins and other molecular chaperones. Elevated *O*-GlcNAc levels prior to heat stress causes increased HSP40 and HSP72 expression, which help to maintain protein folding and solubility during periods of stress (72). Additionally, NMR studies comparing *O*-GlcNAcylated and phosphorylated peptides found that the larger *O*-GlcNAc moiety destabilized α-helicies found in the phospho-peptides, and instead induces a bend (73).

## O-GlcNAc Crosstalk With Other PTMs

The addition or removal of *O*-GlcNAc can alter a variety of biological activities. These changes often occur in concert with other PTMs in an inhibitory or promotive manner. This crosstalk phenomenon helps regulate the dynamic nature of cellular signaling in response to nutrient status. For a more detailed summary of *O*-GlcNAc cross talk with other PTMs, refer to the references (64, 74, 75).

The most direct form of crosstalk is *O*-GlcNAc’s relationship with phosphorylation. This is because both are serine and/or threonine modifications and, therefore, have the potential to compete for the same or proximal sites. This inhibitory form is referred to as reciprocal crosstalk. An example of this can be seen in the oncoprotein c-Myc at thr58, regulating its transactivation (76–78). Crosstalk PTMs more commonly occurs at a distance rather than at the same site. This can occur on near-by amino acids or at a distance, referred to as proximal or distal crosstalk, respectively. For these types, *O*-GlcNAcylation can either promote or inhibit the addition of subsequent PTMs. *O*-GlcNAcylation of p53, a tumor suppressor gene, at ser149 blocks its phosphorylation at thr155, which is known to induce its ubiquitin-proteasomal degradation. As a result, p53 accumulates in the cytoplasm, allowing for increased apoptotic activity (**Figure 1**) (79).

Furthermore, different PTMs often regulate each other’s cycling enzymes. Microarray studies have indicated that over 80% of the human kinome are substrates for OGT (53) and a search of the https://www.oglcnac.mcw.edu/statistics/ yields over 700 results for *O*-GlcNAcylated kinases as of November 2021. These modifications have been known to both inhibit and promote enzymatic activity and even change their substrate specificity, as is the case for Casein kinase 2α (CK2α). *O*-GlcNAcylation at ser347 blocks its phosphorylation at thr344. These two modified CK2α enzymes were screened against a protein microarray and found to have different substrate specificity (80).

Additionally, tyrosine phosphorylation of OGT, resulting from stimulation of the insulin receptor, shows increased transferase activity (81). *O*-GlcNAcylation of other PTM cycling enzymes is also known to regulate activity. *O*-GlcNAc modification of the histone lysine methyltransferase MLL5 was demonstrated to promote methylation of H3K4, triggering cell lineage determination in HL60 lymphocytes (82).

## Nutrient Sensing

While the majority of cellular glucose uptake is directed to glycolytic pathways primarily used for energy generation and storage, about 2-5% is processed through the hexosamine biosynthetic pathway (HBP) (15). The formation of the final product in this pathway, alpha uridine diphosphate-N-acetylglucosamine (UDP-GlcNAc), combines to monitor the metabolism of glucose, amino acids, fatty acids, and nucleotides. Consequently, the cellular concentration of UDP-GlcNAc is responsive to nutrient levels and flux through the pathway (83, 84). This product is either transported to the ER and Golgi for constructing extracellular, endomembrane oligosaccharides or remains in the nucleus and cytoplasm as the donor for O-GlcNAcylation (1, 85, 86).

The intracellular UDP-GlcNAc levels are known to affect OGT’s activity and substrate specificity. This was demonstrated in 1999 by Hart and Kreppel using recombinant OGT in *in vitro*-based experiments comparing its activity and affinity for different peptide substrates under various UDP-GlcNAc concentrations. Remarkably multiple apparent K_m_ values were found by varying UDP-GlcNAc concentrations for different substrates. Furthermore different substrates exhibited varying degrees of GlcNAcylation over a range of UDP-GlcNAc concentrations, indicating that *O*-GlcNAcylation is highly responsive to nutrient levels in a substrate specific manner (11). These early experiments have since been demonstrated in both cell and animal models, particularly in relationship to hyperglycemia or nutrient deprivation (87–89).

**Figure 2:**
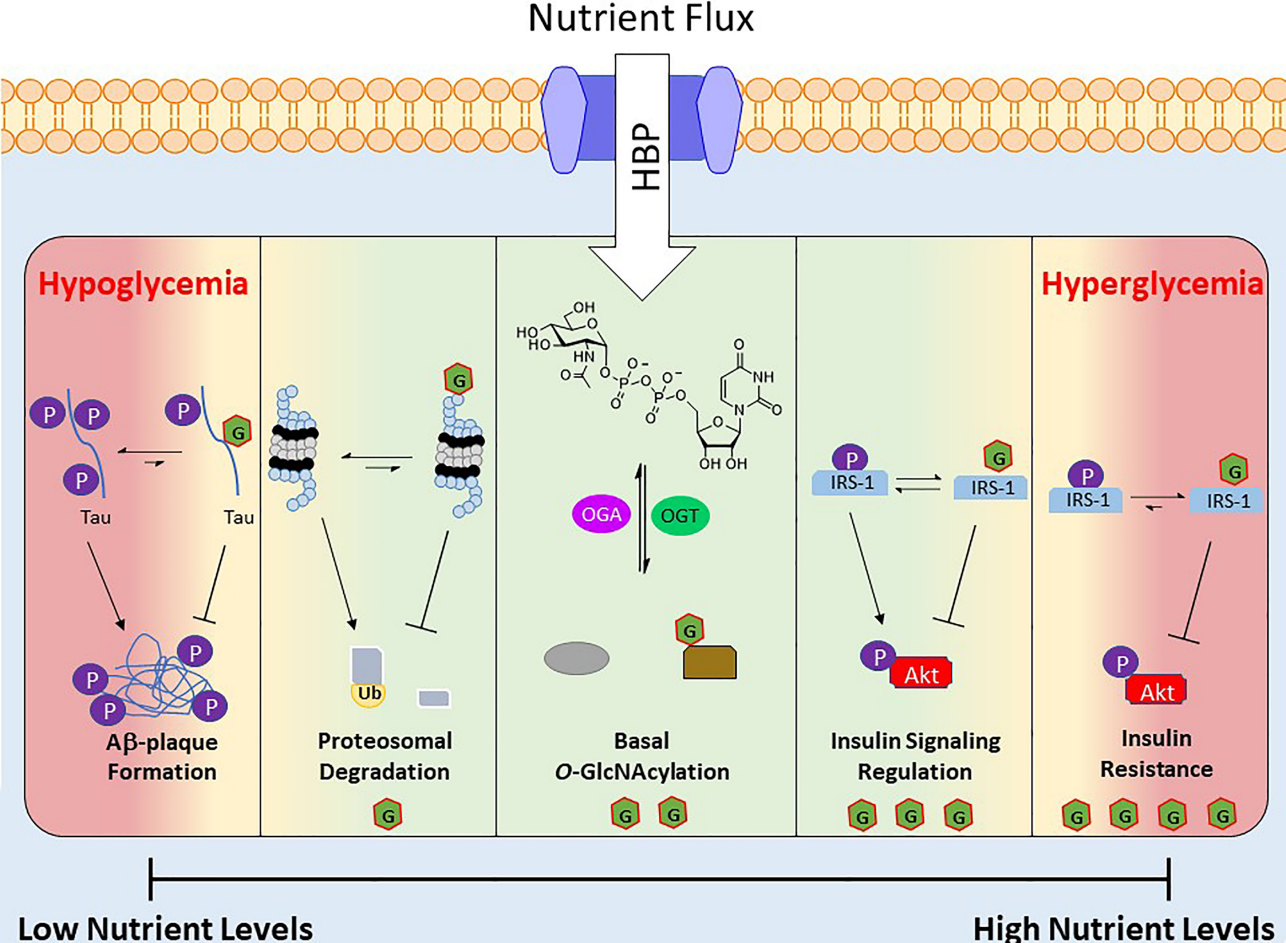
Depiction of *O*-GlcNAc’s role as a nutrient sensing rheostat. Nutrient flux directly impacts the levels of UDP-GlcNAc, which regulates the activity and substrate specificity of OGT. As a result the *O*-GlcNAcylated proteins and their corresponding biological functions are a response to the nutrient levels. Below are several examples of *O*-GlcNAc’s effect at various nutrient levels. Green area is “normal” effects of O-GlcNAcylation and the red areas are O-GlcNAcylation’s effects under extreme nutrient levels.

The nutrient status of a cell and the corresponding levels of UDP-GlcNAc and subsets of *O*-GlcNAcylated proteins act as a nutritional fingerprint imparting changes in biological processes, resulting in nutrient-based phenotypes. Due to this relationship *O*-GlcNAc is often referred to as a nutrient sensing rheostat (**Figure 2**). For example, *O*-GlcNAcylation of the proteasome subunit 19S is correlated with reduced activity (90), which has been demonstrated in the degradation of Sp1 transcription factor under nutrient deprivation (88). This increase in proteasome activity under reduced nutrient levels has been speculated to serve as a catabolic mechanism to regulate amino acid availability (54). Similarly *O*-GlcNAc has been shown to regulate cellular processes under increased nutrient levels, an example of which occurs in the insulin signaling pathway. Upon insulin stimulation, glucose uptake is increased and a signaling cascade initiates various processes (glycolysis, glycogen synthesis, lipogenesis). One of the early stages of this pathway is the phosphorylation of insulin receptor substrate 1 (IRS-1), which induces its association with PI3K and further propagates the signaling cascade towards Akt phosphorylation and activation (91). Various groups have shown that IRS-1 is *O*-GlcNAcylated upon insulin stimulation and that increasing this PTM, *via* OGA inhibition or OGT overexpression, reduces its association with PI3K, attenuating insulin signaling (92–94).

Although *O*-GlcNAc acts to regulate cellular functions during alterations in nutrient levels, prolonged extremes of nutritional flux are linked to disease states. For example, hyperglycemia in type 2 diabetes mellitus is associated with consistently elevated levels of *O*-GlcNAc (16). Similarly OGT overexpression or OGA inhibition has been shown to induce insulin resistance in various cell and animal models (95, 96), and insulin sensitivity is improved *via* OGT KO in mice (97). At the other end of the spectrum is hypoglycemia, which is consequently liked to decreased protein O-GlcNAcylation, is a common feature of many neurodegenerative diseases such as Alzheimer’s disease (AD) (98, 99). Studies in this area have found that hyperphosphorylation of β-amyloid precursor protein (APP) and Tau induces Aβ plaque formations, but *O*-GlcNAcylation of these proteins prevents this aggregation (**Figure 2**) (100).

## *O*-GlcNAc’s Involvement in the Immune System

As described above, *O*-GlcNAc is extensively involved in a multitude of cellular processes, and the immune system is no exception. Many reviews have been written detailing *O*-GlcNAc’s role in various immune system aspects, such as: T-cell development (65), inflammation (101), infection (102), autoimmunity (103), lymphocytic cancers (104), immunometabolism (105), and broad overviews (106). In this section a brief overview of several aspects will be discussed highlighting *O*-GlcNAc’s function in the immune system, particularly as it relates to sensing nutritional cues that govern protein activities.

There are several instances during lymphocyte development and activation that requires a metabolic shift from relying on oxidative phosphorylation to glycolysis and glutaminolytic metabolism. In these situations, glucose and amino acids, particularly glutamine, uptake is dramatically increased, the latter of which leads to increased fatty acid and cholesterol synthesis (107–109). This influx in metabolites is necessary to fulfil the needs of increased cell growth and proliferation required in T-cell development (65) and lymphocyte activation (110). Accompanying this increase in nutrient flux is an increase in UDP-GlcNAc and *O*-GlcNAcylated protein levels (111), which have been found to impart signaling cues important to the required cellular process. One of the most studied of which is T-cell differentiation. Following TCR activation, T cells undergo several stages ultimately becoming various effector lineages based on its microenvironment and signaling. Increased *O*-GlcNAc levels were found during the transition from DN3 to DN4 stage and during positive selection, corresponding to increased glucose and glutamine uptake. In these studies, *ogt* knock out just before the DP stage in mice, diminished the population of mature T cells (112). *O*-GLcNAc has also been shown to be key in B cell survival and activity. Conditional *ogt* allele deletion at various stages of B cell development in mice showed increased apoptosis in mature B cells *via* defects in BAFFR mediated pathways, indicating OGT’s importance in maintaining homeostasis. The same study also showed that OGT KO had reduced B-cell activation corresponding to a decrease in NF-κB nuclear translocation (113).

While sufficient nutrient flux is important to survival, differentiation, and activation in immune cells, chronic aberration of nutrients levels are known to be detrimental. Hyperglycemic conditions, as in diabetes, increases the *O*-GlcNAcylation and activation of the nuclear factor kappa-light-chain-enhancer of activated B cells (NF-κB), similarly increasing the production of pro-inflammatory cytokines (114, 115). Hyperglycemia has also been shown to alter the polarization of macrophages to the less immunogenic M2 state, which correlated with tumorgenicity, suggesting a possible mechanism of immune system evasion (116).

One of the earliest indications of *O*-GlcNAc’s impact on the immune system came in 1991 from Kearse and Hart who demonstrated that, upon T-cell activation *O*-GlcNAcylated protein density rapidly change from the cytosol to the nucleus in a protein-specific manner (111). These results foreshadowed future research demonstrating *O*-GlcNAc’s regulation of key transcription factors, one of the most studied and significant of which being NF-κB (117). NF-κB is a dimeric protein that regulates cytokine production and cell proliferation, and is key in the activation and maturation of lymphocytes. Upon TCR or BCR-activation, the cytosolic dimer is phosphorylated resulting in the ubiquitination and degradation of its complexed inhibitor, inhibitor of κB protein (IκB). This dissociation event in turn induces nuclear localization of NF-κB and subsequent gene expression (118). *O*-GlcNAcylation of NF-κB’s subunits was found to be paramount for this translocalization process in B and T cells (117). Mutagenesis studies in T-cells revealed that *O*-GlcNAcylation of the subunit c-Rel was directly related to its nuclear localization, promoter binding and gene expression of IL2, IFNG, and CSF2 (119). Interestingly the location of this *O*-GlcNAcylation site is directly adjacent to the IκB binding domain of NF-κB and its phosphorylation was not found to be altered. Other studies performed in epithelial cell lines have demonstrated that *O*-GlcNAc functionalization of NF-κB is not only necessary for its translocation but also helps to mediate acetylation of p65 subunit which increases NF-κB gene transcription. This acetylation results from an interaction with the acetyltransferase CBP/p300, which is weakened by p65 phosphorylation (120). This inhibitory phosphorylation was shown to be blocked by *O*-GlcNAcylation of p65 on thr305 and ser319 (121). *O*-GlcNAc modification of NF-κB has been shown to inhibit activation in various cells. For example, In macrophages NF-κB activation promotes inducible nitric oxide synthase (iNOS) expression which is a key enzyme in the innate immune system for killing infectious bacteria (122). Overexpression of OGT in RAW264.7 macrophage cells and BV2 microglial cells resulted in suppressed iNOS expression corresponding to increased *O*-GlcNAcylation of p65 and c-Rel (123). Supporting this inhibitory effect, OGA inhibition in LPS treated RAW264.7 microglial cells increased iNOS expression (124). These results highlight the fact that *O*-GlcNAc’s regulatory roles are cell specific.

## Discussion

Since its discovery in 1983 (1, 2), the *O*-linked-N-acetylglucosamine monosaccharide post-translational-modification has been demonstrated to be a key modification for regulating and maintaining a variety of biological functions in response to nutrient levels. These processes are controlled by changes in protein function driven by intricate crosstalk between *O*-GlcNAc and other PTMs. Aberrations in nutrient flux and *O*-GlcNAc levels has been demonstrated in numerous disease states. Disease phenotypes can be induced by directly altering *O*-GlcNAc cycling (enzyme inhibition or knockout) or *via* nutrient manipulation, indicating that changes in *O*-GlcNAcylation can have a direct effect on the progression or development of various pathologies.

Most work regarding *O*-GlcNAc in the immunology field has been focused around lymphocyte activation, survival, and development. The majority of these studies focus on macrophages, B cells and T cells. As a result, little is known about *O*-GlcNAc’s role in other immune cells. For example, changes in *O*-GlcNAcylation have been found in natural killer (NK) cells and neutrophils during cytotoxicity events and chemotactic stimulation respectively (125, 126). Furthermore glucosamine supplementation results in increased Rac activity in neutrophils, important for neutrophil mobilization, and decreased cytotoxic activity in NK cells (127, 128). These studies suggest that *O*-GlcNAc is involved in these processes, however specific mechanisms have yet to be determined. Even less work has been done with regards to dendritic cells, eosinophils, and basophils. Considering that *O*-GlcNAc’s effects may be cell specific, recall the effect on NF-κB and pro-/anti-inflammatory responses in various cells, these fundamental gaps in understanding may be significant.

Additionally, *O*-GlcNAc’s role in lymphoid cancers and autoimmune disease remains unexplored. Elevated glucose flux and metabolism is observed in nearly all cancers known as the Warburg effect. Consequently *O*-GlcNAcylated protein levels are also elevated (129). In Chronic Lymphoid Leukemia (CLL) *O*-GlcNAcylation levels of p53, Akt, c-Myc, and STAT5 are increased with respect to normal basal levels, promoting cell proliferation (130, 131). However, OGA inhibition with thiamet G increased cell sensitivity of chemotherapy for human leukemia cell lines (132). Although exciting, these contradictory effects highlight the need for further investigation before they can be applied in therapeutics.

Similar to cancer, increased glucose uptake in immune cells is a key feature of acute inflammation (133). Furthermore, prolonged hyperglycemia observed in type 2 diabetes mellitus is known to lead to chronic inflammation in patients that leads to an increased risk of autoimmunity development (134). A recent study performed by Liu et al. demonstrated enhanced pro-inflammatory cytokine production in virus-challenged primary mononuclear blood cells and pulmonary epithelial cells when treated with OGA inhibitor or glucosamine. These effects were extended to a mouse model and found to correlate with decreased survival rates. Further studies determined OGT’s interaction with interferon regulatory factor 5 (IRF5) as key regulator of the increased cytokine production (135). Conversely OGT was found to be paramount to the lineage stability of regulatory T cells (Treg cells). Ogt knockout in Treg cells in culture and mice exhibited a severe autoimmune phenotype resulting from attenuated IL-2/STAT5 signaling attributed to decreased glycosylation (136). This Ogt knockout-related Treg pathology was also found to exacerbate hepatitis in an autoimmune rat model (137). Taken together *O*-GlcNAc seems to play different roles in different aspects of autoimmunity. More studies are required to fully detail the relationship between aberrant nutrient flux, *O*-GlcNAcylation, and autoimmunity.

## Author Contributions

MM: Wrote the article and made the figures. GH: Edited the article and gave direction for sections. All authors contributed to the article and approved the submitted version.

## Funding

This work was supported by National Institutes of Health Grants R01DK61171 and R01GM116891.

## Conflict of Interest

The authors declare that the research was conducted in the absence of any commercial or financial relationships that could be construed as a potential conflict of interest.

## Publisher’s Note

All claims expressed in this article are solely those of the authors and do not necessarily represent those of their affiliated organizations, or those of the publisher, the editors and the reviewers. Any product that may be evaluated in this article, or claim that may be made by its manufacturer, is not guaranteed or endorsed by the publisher.
